# Guardians and research staff experiences and views about the consent process in hospital-based paediatric research studies in urban Malawi: A qualitative study

**DOI:** 10.1186/s12910-022-00865-x

**Published:** 2022-12-05

**Authors:** Mtisunge Joshua Gondwe, Neema Mtunthama Toto, Charity Gunda, Markus Gmeiner, Ian J. C. MacCormick, David Lalloo, Michael Parker, Nicola Desmond

**Affiliations:** 1 Malawi-Liverpool-Wellcome Trust Clinical Research Programme, Blantyre, Malawi; 2 Liverpool School of Tropical Medicine, Liverpool, UK; 3 The Ethox Centre, University of Oxford, Oxford, UK; 4 Radboud University Medical Centre, Nijmegen, The Netherlands

**Keywords:** Paediatric informed consent, Acute setting, Non-acute setting

## Abstract

**Background:**

Obtaining consent has become a standard way of respecting the patient’s rights and autonomy in clinical research. Ethical guidelines recommend that the child’s parent/s or authorised legal guardian provides informed consent for their child’s participation. However, obtaining informed consent in paediatric research is challenging. Parents become vulnerable because of stress related to their child’s illness. Understanding the views held by guardians and researchers about the consent process in Malawi, where there are limitations in health care access and research literacy will assist in developing appropriate consent guidelines.

**Methods:**

We conducted 20 in-depth interviews with guardians of children and research staff who had participated in paediatric clinical trial and observational studies in acute and non-acute settings in the Southern Region of Malawi. Interviews were audio-recorded, transcribed verbatim, and thematically analysed. Interviews were compared across studies and settings to identify differences and similarities in participants’ views about informed consent processes. Data analysis was facilitated by NVIVO 11 software.

**Results:**

All participants across study types and settings reported that they associated participating in research with therapeutic benefits. Substantial differences were noted in the decision-making process across study settings. Guardians from acute studies felt that the role of their spouses was neglected during consenting, while staff reported that they had problems obtaining consent from guardians when their partners were not present. Across all study types and settings, research staff reported that they emphasised the benefits more than the risks of the study to participants, due to pressure to recruit. Participants from non-acute settings were more likely to recall information shared during the consent process than participants in the acute setting.

**Conclusion:**

The health care context, culture and research process influenced participants’ understanding of study information across study types and settings. We advise research managers or principal investigators to define minimum requirements that would not compromise the consent process and conduct study specific training for staff. The use of one size fits all consent process may not be ideal. More guidance is needed on how these differences can be incorporated during the consent process to improve understanding and delivery of consent.

*Trial registration* Not applicable.

**Supplementary Information:**

The online version contains supplementary material available at 10.1186/s12910-022-00865-x.

## Background

Informed consent is the cornerstone of ethical biomedical research involving humans [1, 2]. Obtaining consent has become a standard way of respecting the patient’s rights and autonomy in clinical research [3]. The Declaration of Helsinki [4] and the Council for Information Organisation of Medical Sciences (CIOMS) guidelines [5] describe the process of obtaining informed consent and the importance of the participants’ understanding of information before enrolment in research. However, the informed consent process remains a contentious issue in both clinical and public health research conducted in limited-resource settings [2, 6]. There is still uncertainty about the adequacy of informed consent procedures [2, 7]. Patients’ lack of comprehension of presented information about the research is an obstacle to informed consent which may be due to unfamiliar terminology, hospital environments, cultural and language barriers, and low literacy levels [3, 8–10]. It has been reported that participants who have given their consent do not fully understand their rights as participants and a significant proportion of them do not remember consenting to participation on their own or on behalf of their child [11]. In systematic reviews [12, 13], participants from low-middle income countries (LMICs) and those with a low level of literacy were less likely to understand some aspects of the study information during the consenting process such as randomisation, voluntariness, and right to withdraw.

There remains a high disease burden in Sub-Saharan Africa, with 1 in 13 children dying before their fifth birthday [14]. As such, there is a need for more research that directly benefits children in low-income settings. While medical research in children is vital, children need special protection as they are less likely to express their needs or defend their interests compared to adults [15]. Studies from Kenya, Uganda and Malawi have reported challenges surrounding informed consent processes in paediatric research in low-income settings [8, 9, 16, 17]. These challenges include lack of familiarity with and misunderstanding of research concepts (randomisation, placebo), local beliefs, education level, parents’ interest, unclear difference between research and therapeutic investigation. In research involving children, the child’s parent/s or authorised legal representative is required to provide informed consent for their child’s participation [18]. Parental Consent is obtained when a child or minor aged 0–7 years is included in research [19]. The parent/guardian must sign a parental permission consent document on behalf of the child [19]. If a child aged 7–17 years is included, ‘Assent’ must be obtained in addition to parental consent [19]. Assent is a child’s affirmative agreement to participate in research, meaning both the child and their guardian must give permission [19]. The parents of children become vulnerable to consent as they are preoccupied with the child’s illness [20] and this might affect their understanding and decision to enroll their child in the study. Parental understanding is often influenced by educational status, religious, cultural beliefs, and family or peer pressure [21]. Studies in LMICs, such as Uganda, Swaziland, Pakistan, and Mali have found that comprehension is adversely affected by factors such as high illiteracy levels, the high social status of physicians, and poor access to medical services, which affects understanding during the consenting process [6, 22–24]. Furthermore, many parents in low-resource settings like in Mali and Malawi, struggle to differentiate between medical research and routine clinical care due to low research literacy and lack of understanding of research terminology, which compromises their understanding of informed consent. The guardians may think that participating in research will result in medical benefits to the participant [17, 24].

Obtaining meaningful informed consent from study participants requires accurate presentation of study information in addition to a well-written consent form [25]. Very few researchers have tested the understanding of participants throughout trials conducted in low-income settings [1, 8–10, 26]. In Kenya, a study looking at the understanding of informed consent among participants in paediatric case studies found inter-related issues (conceptual and linguistic barriers, the critical and complex role of communicators in consent procedures, research unit and community relations, and sensitive issues such as blood sampling) that need to be considered [9]. A few studies have evaluated the repeated assessment of comprehension in relation to informed consent and the challenges of consenting in HIV/AIDS-related trials in sub-Saharan Africa [8, 27, 28]. Other researchers have recommended incorporating assessment of participants’ understanding throughout the study into research projects [8]. However, it was also reported that, during the consenting process, research staff emphasise issues of the trial that they anticipate a participant will understand easily, and do not attend as much to issues that might be difficult for a participant to understand such as study design, randomisation, and the selection procedure [29]. This also affects informed consent, as participants are partially informed.

Little has been published in the literature on assessing understanding of participants on the informed consent process within specific study designs. Looking at different study designs will assist in understanding informed consent processes. Studies have reported challenges in recruiting and obtaining consent from participants to participate in emergency settings due to stress they might be experiencing [30]. Recently, a study of caregiver and provider experiences in research conducted in Malawi confirmed how challenging obtaining informed consent was in a paediatric critical care unit [17]. Barriers included inadequate time to handle patient care and research related tasks, misunderstanding of research goals, parental stress due to their child’s illness, social structure, and community doubt about research procedures [17]. The study found that caregivers’ understanding of informed consent did not appear to be affected by poor comprehension and low educational levels [17]. The study further suggested ways to improve the consent process such as greater community involvement and use of patient advocates to champion the consent process [17]. Another study conducted in Malawi, which explored the effectiveness of a rectal antimalarial in children with moderately severe malaria found that parents with severely ill children struggled to understand large amounts of information due to the stress they encountered based on the child’s condition [31]. A rural Ghanaian genomics study (“MalariaGEN”) also highlighted the complexity of seeking consent in emergency research situations. The researchers felt that it was “practically impossible and ethically inappropriate to conduct a detailed consent process for research before collecting the samples needed for diagnosis and treatment” [25]. Willingness to participate in the study is influenced by the enhanced medical care the participants receive in the studies [32, 33].

Low levels of awareness of human and medical rights highlights the importance of understanding consent in LMICs such as Malawi [34]. This study aimed to evaluate participants’ understanding of study information by comparing the effect of study design and research setting. It also aimed to understand the challenges faced by research staff around the informed consent processes. The findings contribute to the strengthening of the practical application and quality of consent locally, as well as to global research on paediatric consent processes.

### Context

This study was conducted in the paediatric department and paediatric research wards at Queen Elizabeth Central Hospital (QECH) where Malawi-Liverpool-Wellcome Trust Clinical Research Programme (MLW) conducts paediatric clinical research. Queen Elizabeth Central Hospital is the largest referral hospital in the southern region of Malawi, offering speciality paediatric care for admission and outpatient services to children aged 0–14 years. The primary criterion for including a specific clinical trial or observation study was (1) the study was recruiting paediatric patients aged between 2 months to 7 years and (2) the study was taking place in an acute or non-acute setting in the QECH paediatric department. For this study, acute is defined as inpatient care. The acute setting is where patients remain under constant care often with emergency conditions [35]. Non-acute refers to outpatient care, where a clinic or medical setting typically deals with non-emergency conditions [35]. We recruited study participants from one clinical trial and two observational studies conducted in an acute setting and one clinical trial and two observational studies in a non-acute setting.

The interventional study in the acute setting was the “TRansfusion and TReatment of severe Anaemia in an African Children Trial” (TRACT). The study was an unblinded randomised controlled trial, and included children aged 2 months to 12 years admitted to hospital with severe anaemia. Children were allocated into three groups (study product, routine care and no intervention) [36]. The interventional study in the non-acute setting was the “bronchopulmonary function in response to azithromycin treatment for chronic lung disease in HIV-infected children trial” (BREATHE). The study was a double-blinded trial, and included children aged 6–19 years with HIV-associated chronic lung disease who have been receiving Antiretroviral therapy. Half of the children received the study product and half received the placebo [37].

The observational study in the acute setting was from the vaccine surveillance (VACSURV) programme of studies; the first study examined pneumococcal carriage among vaccinated, sick children aged 1–5 years admitted at QECH; and the second study examined rotavirus carriage among vaccinated children who presented with diarrhoea at QECH. The observational study in the non-acute setting was Pneumococcal Carriage in Vulnerable Populations in Africa (PCVPA). It examined pneumococcal carriage among non-vaccinated healthy school children aged 5–10 years and healthy vaccinated children aged 3–4 years of age. The second observation study in the non-acute setting was a Typhoid study. The study examined Salmonella Typhi bacteraemia carriage in young children presenting with fever with a negative Malaria test.

## Methods

This cross-sectional study used a descriptive qualitative approach to explore guardian and staff experiences and views about the consent process within MLW paediatric studies conducted at QECH. The study covered an 8-month period from September 2017 to April 2018.

We conducted a total of 20 In-Depth Interviews (IDIs) with purposively selected study staff (research nurses) and guardians of children who were involved in paediatric clinical research at QECH. We recruited participants from four types of study to allow for a comprehensive understanding of views on the effects of study design and setting on participants’ understanding of study information during the consent process. We conducted four IDIs each with guardians of children who took part in (1) clinical trial, acute setting, (2) clinical trial, non-acute setting, (3) observational study, acute setting, and (4) observational study, non-acute setting. We also conducted IDIs with study staff who were extensively involved in obtaining informed consent. In each study type, one staff participated in an IDI (4 in total).

Permission was obtained from all investigators of clinical trials and observational studies to recruit their participants. For guardian IDIs, we approached participants who were enrolled in clinical trials and observational studies at the time of our study. Research staff from participating studies informed study participants about this study and briefed them about our study objectives during the consent process. Those interested in participating in our study were referred to our research assistant who approached the participants on the same day and gave detailed information about our study. Voluntary consent was obtained, and an IDI conducted within 72 h of being consented to participate in the main study. In-depth interviews for acute setting participants were conducted while still in hospital. In-depth interviews for non-acute setting participants were conducted in hospital or at patient homes. To recruit research staff, respective principal investigators briefed their staff about the study during team meetings. We recruited only research nurses, who confirmed their involvement in obtaining informed consent in the study. Following their willingness to participate in an IDI, research nurses were called for an interview on an agreed date. Table 1 summaries characteristics of study participants.

**Table 1:** Characteristics of study participants

Study type	Participants		Number of IDIs	Total number of participants
	Guardians	Staff		
Clinical trial, acute setting	4	1	5	5
Clinical trial, non-acute setting	4	1	5	5
Observational study, acute setting	4	1	5	5
Observational study, non-acute setting	4	1	5	5
Totals	16	4	20	20

In-depth interviews were guided by open ended questions based on an interview guide (Additional file 1: Appendices 1 and 2) informed by the study objectives, a literature review, and an iterative analysis approach where revisions were made considering emerging findings. The interview questions were piloted to first four participants (one per study design) and the transcripts were not included in the final analysis. Questions were revised based on participants’ experiences and responses that required further exploration. Data collection for guardian IDIs was stopped when saturation was achieved. Achieving saturation was determined when iterative analysis led to no further adjustments to the topic guides, and no novel codes emerging [38, 39]. For staff IDIs, we interviewed research staff actively involved in obtaining informed consent within each study.

Every interview was recorded and transcribed verbatim in either Chichewa or English, depending on whether it was conducted entirely in that language or not. The interview transcripts were analysed thematically using the method of constant comparison. With this method, MJG read the individual interview scripts repeatedly before comparing issues and experiences (i.e., themes), which cut across different accounts. A comparative analysis of guardian and staff accounts was also undertaken to identify differences and similarities in their views about informed consent processes. Interview transcripts were coded inductively. Themes emerging from the data were discussed among the investigators and coding differences were resolved by reaching a consensus. A coding framework was then developed which reflected these themes. Table 2 summarises themes generated from the analysis. As part of the data analysis process, the qualitative analysis software package NVIVO 11 (QSR International) was used to facilitate data coding and retrieval.

**Table 2:** Summary of three major themes and its subthemes derived from the interviews

Themes	Subthemes
Decision making	Benefits
	Family role in decision making
Challenges with consent process	Timing of consent
	Privacy during consent
	Understanding and Comprehension of consent forms
Suggestions to improve consent process	Consent process
	Community sensitisation

Ethics approval was received from the Malawi College of Medicine Research Ethics Committee (P.04/15/1719) and Liverpool School of Tropical Medicine Research Ethics Committee (14-060). Institutional and study permission were sought from QECH as the participating institution and all study principal investigators. In addition, their letters of support were submitted to the ethics committee prior to interviews. All participants gave their signed or thumb-printed written informed consent to take part in an interview.

## RESULTS

### Overview

Study participants shared diverse views regarding the understanding of study information, views about the mode of administration, timing of informed consent and challenges during informed consent processes. Views from guardians and research staff are combined since there are similarities that need to be reported simultaneously. These views are compared across all four study types (clinical trial and observational; acute and non-acute settings). In this section we present our findings under three key themes and the eight sub-themes that emerged (Table 2).

### Decision making

#### Benefits

Participants’ motivation to participate in the study varied across study designs and settings and was mainly influenced by the benefits they experienced or anticipated to experience after enrolling in the study. Benefits included treatment, attention, awareness, and knowledge. However, the types of personal benefits described by participants slightly differed by study setting. Guardians from both clinical trials and observational studies in acute settings described treatment and care the child receives when enrolled in a study as better than routine care. Participants from the clinical trial in the acute setting explained:

“My motivation to participate in this study was due to frequent child assessments by the study team. I had been in different hospitals, but the type of care I had been receiving was exceptional. The way they received me and the care they gave my child was more than what I can get in private hospitals.’’ [Clinical trial, acute setting, Guardian 3].

“They explained to me that if I would enrol in this study, I would receive the best treatment. The way the child was, I thought if I refused, my child could have died, it was better for me to agree to participate and save the life of my child.” [Clinical trial, acute setting, Guardian 2].

Another participant from observational studies in acute settings added:

“…the benefit is that they will be screening the child frequently during the study.’’ [Observational study, acute setting, Guardian 10].

Guardians from clinical trials and observational studies in both acute and non-acute settings reported that there is more information provided about the child’s medical condition when participating in a clinical study as explained below:

“I had accepted to participate in this study because I wanted to know what disease condition was troubling my child.’’ [Observational study, acute setting, Guardian 9].

“…I was able to understand my child’s problem through participating in this study.” [Clinical trial, non-acute setting, Guardian 6].

“I really wanted to find out what was the problem with my child. They found him with malaria. He received treatment but did not improve. This was the reason why I had decided to join this study so that I should find out what was the cause.’’ [Observational study, non-acute setting, Guardian 13].

In addition, checking the well-being of each participant by the study team during home visits was reported as a benefit by guardians from the clinical trial and observational study in the non-acute setting. Participants valued these visits and felt respected.

“We have been helped a lot after enrolling in the study. For example, the study team made frequent follow-ups at home. If a child was sick, they provided transport to go to hospital. Sometimes they came and picked us from home. If admitted, they would come and see the child as their relative.’’ [Clinical trial, non- acute setting, Guardian 8].

Research staff from an interventional study in an acute setting and observation studies in acute and non-acute settings concurred with what participants narrated as motivators to participate in the study. According to the research staff an expectation of special care when enrolled in a study is a common motivator for participation in paediatric studies. The research staff felt that this has a tremendous effect on participants’ willingness to participate.

“When the parents came to the hospital with sick children, they consented because they wanted the child to get prompt treatment. After treatment, they might refuse to continue with the study, miss follow-ups or finish the study without knowing what the study is all about.” [Clinical trial, acute setting, Research Staff 1].

“The majority of participants thought that if they joined a study, they were going to be treated well, given the better attention than those who have not joined the study. This was worrisome because the mothers did not know why they had joined the study and the type of the study.” [Observational study, acute setting, Research Nurse 3].

#### Family role in decision-making

Guardians from clinical trial and observational studies in acute settings felt that the role of their family members was neglected during consenting, while staff reported that they had problems obtaining consent from guardians when their spouses or family members were not present. Guardians from clinical trials and observational studies from acute settings expressed a concern that their spouses or partners did not take part during the consenting process as they were not present at the hospital.

“I decided without my husband. It would depend on how I would explain it to my husband. However, I might also face a problem with him.” [Clinical trial, acute setting, Guardian 2].

“My husband was not involved when I was deciding to enrol the child in this study. I did not discuss it with him. I did not know if he would accept this or not.” [Observational study, acute setting, Guardian 10].

This experience was different for guardians participating in clinical trial and observational studies in non-acute settings, where decisions on participation were less immediate.

“I was given a chance to consult other family members before deciding. However, the mother of the child abandoned the child when she was one year old, so I am the mother, father, and grandmother of this child. So, I just decided on my own.” [Clinical trial, non-acute setting, Guardian 5].

In addition, research staff from clinical trials and observational studies in both acute and non-acute settings shared their experiences with obtaining consent from guardians in the absence of their spouses or partners.

“Sometimes the mother finds it challenging to provide her consent because she looks to her spouse or other family members for approval. Most of the time, when the mother gave her consent while her husband was away, the husband was usually uninformed of the consent when he arrived at the next visit with a child, and mostly they withdraw participation. When you follow them in the community, they shun away from you. I believe that all key guardians should be involved in the consenting process.” [Clinical trial, acute setting, Research staff 1].

“We gave them a chance and time to go back home to discuss with the husband before deciding. First, we did verbal consent. If the husband refused the first time, and they had come the second time we emphasised finding out if they had agreed with the husband. We even allowed the husband coming along with them next time. After they have decided as the family, we did full consent again. This eased the consenting process, and it was also the right way of ensuring that full informed consent was obtained.” [Clinical trial, non-acute setting, Research staff 2].

“In my experience, most guardians in the ward were mothers, and it was difficult for them to decide. Although studies allowed involving the husband in making the decision, most of the women did not come back if given a chance to inform the husband, others they said my husband had refused as an excuse for them not to participate. Most times, we took consent from mothers, and we just informed them to tell the husband. However, I have noted that when the husband was around, it was easier to recruit participants than when only the mother.” [Observational study, Acute setting, Research staff 3].

### Challenges with the consent process

#### Timing of consent

Research staff from an observational study in non-acute setting shared their experience on time available for consent:

“I felt we spent less time with participants during consenting. Our study did not have a study clinician, and we used government clinicians. So, we met with clients after they had spent even more than 2 hours with clinicians, and they were due to receive treatment. So, most of the time we rushed through the consenting process because we did not want to disturb the queue.” [Observational study, non-acute setting, Research staff 4].

The guardians and study staff were asked about the duration and timing of the approach for informed consent (see Additional file 1). Guardians from acute settings, raised concerns over insufficient time and inappropriate timing for consent:

“They did not give me enough time. Staff should have enough time to ask the participant if ready to join their study and give a chance to the participant to discuss with her family before deciding otherwise. The time was not enough for me.” [Observational study, acute setting, Guardian 10].

“They communicated to me about this study before my child received treatment. It was not a good time since my child was having convulsions, and they spent a few minutes with me which was not enough time for me to decide.” [Clinical trial, acute setting, Guardian 4].

While guardians from the clinical trials and observational studies in acute settings shared their negative experiences with time allocation and timing, the guardians from the clinical trial in a non-acute setting described positive experiences:

“They gave us enough time; they ask you if you have finished with hospital routines for that day or if you had something to do on that day. They allowed you to do all your programmes then when you finished, it was when you could meet with them. They did not force you to start with them, but they waited for you.” [Clinical trial, non-acute setting, Guardian 6].

#### Privacy during consent

Research staff were asked if the space they used to obtain consent from participants provided adequate privacy. The research staff from the clinical trial in an acute setting and observational studies from both acute and non-acute settings expressed concerns over privacy during the consent process. Inadequate privacy jeopardised individual consent as peer influence was observed.

“It was challenging doing consent in the High Dependency Unit (HDU) when the child was very sick; you could sit in one corner, while others were still carrying their duties in the same room. Some mothers gave false information to others, and you just find out that they were not coming for follow-ups because they have been discouraged by their friends.” [Clinical trial, acute setting, Research staff 1].

“Privacy was one of the challenges we met during consent. We did not have rooms to use for consenting. We did consent at the bedside. We got distractions most of the time when consenting at the bedside. Sometimes the participants were influenced by friends. If another mother next to bed said this study is good, most mothers would enrol; if said this study is bad, the majority would not enrol.” [Observational study, acute setting, Research staff 3].

“We did not have our own office or room. We always borrowed a room from the Out-Patient Department (OPD). Sometimes they kicked us out of the room anytime during the consenting process, this compromised the privacy of the participants.” [Observational study, non-acute setting, Research staff 4].

Responses from guardians regarding privacy varied considerably. While some complained about it, others accepted it as a normal process:

“I felt uncomfortable discussing my child’s participation in the study on an open space while others were listening. But I could not do otherwise since, I was alone to take care of the child and my child was on oxygen and I could not take him to another room either leave him alone or with somebody” [Observational study, acute setting, Guardian 10].

“I did not see any problem discussing the study while others were listening. We usually discuss in open space about child’s care with nurse and doctors. What I cared most was for my child to get the best care” [Clinical trial, acute setting, Guardian 3].

#### Understanding and comprehension of consent forms

Most guardians across all study types and settings were satisfied with the study information that research staff communicated to them during the consent process. The guardians described that they were able to understand information regarding benefits of the study, follow-up care, and study procedures. However, some guardians from a clinical trial and an observational study in the acute settings expressed concern with understanding or recalling the information shared with them during the consent process regarding risks, voluntariness, study type, randomisation, and treatment group allocation in the clinical trial. The concerns voiced by guardians were related to poor communication and parental distress:

“They explained to me about the pneumonia study, but they did not explain to me adequately, and I had forgotten information they told me, may be because my child was too sick for me to listen to them.” [Observational study, acute setting, Guardian 9].

“What I knew was that they were studying anaemia… About different groups, I have forgotten maybe because my child was too sick during that time… About the type of treatment, they did not tell me.” [Clinical trial, acute setting, Guardian 1].

“They explained to me that I need to participate in this study because my child fell sick frequently. Participating in this study will help my child to be well. The staff did not inform me that I had a right to withdraw at any point in the study.” [Observational study, acute Setting, Guardian 12].

“They did not explain to me about the risks of this study. However, I have been told to come back after two weeks, maybe they will explain that time.” [Clinical trial, non-acute setting, Guardian 6].

When research staff were asked about communicating study information, one of the staff described experiencing challenges in communicating some of the study information due to the demands of other research activities necessary during recruitment:

“Most of the time, we overlook the risks of the study when consenting a participant. We do not stress much about the risks, but we stress much about the benefits. If the study involves drugs, we do not also stress much about the side effects of the drugs. We are supposed to explain properly about the risks but maybe because we are in a rush to recruit more hence omitting other aspects.” [Clinical trial, non-acute setting, Research staff 2].

Research staff also shared their concerns about difficulties engaging with guardians when communicating study information which impacted guardians’ understanding and comprehension of study information. Staff complained that most mothers did not show interest in the conversation, and they just claimed that they had understood the information, but they had not. The staff also complained that only a few mothers collected the information sheets when offered and only a few appeared to read the information sheet.

“Most women do not read the information sheet despite being literate. Sometimes I emphasise that if they have questions, they should call me, but none has ever called me. If they are asking questions, it is about the volume of blood not details of consent. Sometimes if you tell them to read information, they just say read for us, or sometimes leave them on the chair. However, men are the ones that are interested in reading the consent, and they do ask a lot of questions.” [Observational study, non-acute setting, Research staff 4].

When guardians were asked about the study information sheet, some complained that they did not receive one from research staff, while the majority of those who did receive one, accepted that they did not read it because it was lengthy, a repetition of what has been told during the consent process, or they had inadequate time.

“I was not even given a study information leaflet, what I remember is that I signed a paper and gave them back.” [Clinical trial, acute setting, Guardian 1].

“…they also gave me information sheet, but I did not read it as I was still busy with my child and the form was long.” [Clinical trial, acute setting, Guardian 3].

“I was given information leaflet, but I did not read it because I believed that what was in that paper was everything, they have told me.” [Observational study, acute setting, Guardian 12].

### Suggestions to improve the consent process

#### Consent process

The study explored what guardians and research staff regarded to be the most appropriate approaches for obtaining informed consent in different study types and settings. Following concerns raised about insufficient time allocation and inappropriate timing in acute settings and observation studies in a non-acute setting, research staff suggested that the consent process should be flexible and not a one-off activity during the study but a continuous contact with the guardian to ensure understanding of the study information:

It would have been good if we were given a chance to follow up with guardians after consenting to ensure that they understood what we discussed during consent process and keep reminding them, but our protocol could not allow us to do so.” [Observational study, acute setting Research Staff 3].

“I would suggest following a two-step consent as we had been doing in our study, we started with verbal consent (in brief) when the child is critically ill, followed by a detailed consent when the child is stable.” [Clinical trial, acute setting, Research staff 1].

Guardians and research staff also suggested that the most appropriate time for staff to consent guardians is when guardians are stress-free. This being a time when the child is out of danger or has received treatment.

“They should give us study information when the child has received treatment. If the child is very sick and not yet received treatment, we fail to hear properly all the communication from staff.” [Clinical trial, acute setting, Guardian 4].

“The best time to give consent is when a child has received treatment and is stable. This time, the mother is happy, and she will be able to grab more information.” [Clinical trial, acute setting, Research Staff 1].

Furthermore, to promote voluntary participation, research staff suggested utilizing an individual approach to information provided prior to consenting rather than a group approach:

“What I had observed again was that: it was better to do one to one consenting with individuals, than two of them. When we were doing a group consenting, if one said no, everybody refused and if one said yes everybody accepted” [Observational study acute setting Research staff 3].

In addition to an individual approach being suggested, research staff highlighted the importance of research staff having good communication skills to improve the consent process:

“You need to build rapport with guardian for communication to be effective. I suggest, to start asking if guardian had ever heard about the research, or joined any research, so that you could assess previous knowledge which would guide your approach to consent as others might have had bad experience where studies were not well explained and that might affect their participation in the current research.” [Observational study, acute setting, Research staff 3].

“Be patient with guardians during consent process, give them time and explain in a simple term for them to understand.” [Clinical trial, non-acute setting, Research staff 2].

One of the guardians from an observation study in an acute setting complained about the approach the research staff used. Being her first experience with research, she suggested that clearer and more detailed information about the study, benefits, risks and right to participate or not should be provided:

“When approaching someone new to research like me, the staff could use this approach when explaining ‘We are doing a study for children suffering from pneumonia. These are the benefits and risks. You have a chance to ask questions, and you can participate or not and your decision to participate will not affect child’s treatment’. If they could have communicated like that it would have been good” [Observational study acute setting Guardian 10].

Research staff across all study types and settings also suggested a need for frequent and ongoing training opportunities for staff who are involved in the consent process. Despite general training being offered, staff stressed the need for specific study training for those involved in the study consent process:

“We need frequent refresher training about consent process at least every 6 months or 12 months especially specific to the study we are recruiting from as study differs and approaches also differ.” [Observational study, acute setting, Research staff 3].

#### Community sensitisation

Guardians suggested the use of community sensitisation programmes to reach the community and give information about the research before its commencement. This would help to clear research misconceptions, to promote understanding of research and to reach fathers of children participating in research. The suggested methods included: community meetings using chiefs, radios, schools, health centres, churches, and research clubs.

“The best way is to inform the community about the study through chiefs, community meetings, or giving information in health centres before study commencement. This will help in reaching more fathers, and we will be able to decide before the child is sick.” [Observational study, acute setting, Guardian 9].

“You can also use community research clubs to distribute message about the research as they do with HIV programme in the community.” [Clinical trial, acute setting, Guardian 1].

The research staff from an observational study in an acute setting also stressed the importance of community sensitisation before study commencement:

“We first started our study without community sensitisation; it was like a new thing to the participants. We had problems recruiting. Then we decided to start again with community sensitisation in the community meetings, and we involved the chiefs, and it worked well then.” [Observational study, acute setting, Research staff 3].

## Discussion

This study explored the views of guardians and research nurses about the practical aspects of seeking valid informed consent across different study designs and settings in hospital based paediatric research studies. Our study identified challenges about the informed process that were expressed by both guardians and research nurses in paediatric research involved in consenting. The challenges identified were related to the health care context or environment, culture and research procedures and varied across study types and setting. We found that motivation to participate in the study was influenced by the personal benefits participants anticipated or experienced after enrolment in a study in this context. Misunderstandings of the consent process were associated with peer influence and inappropriate timing of consent in the acute setting. The need to involve the head of the family (men) in decision-making affected informed consent processes. Poor staff communication techniques due to limited time also affected the consent process.

### Health care context or environment

Both staff and guardians who participated in clinical trials in acute and non-acute study settings and observational studies prioritised the ethics of care over research ethics codes of practices. Access to better health care was associated with better health outcomes. Our findings are similar to studies conducted in high-income countries like France and the United States [40, 41]. These studies reported that parents and patients participating in clinical trials who had life-threatening illnesses perceived that enrolment in hospital-based clinical research was beneficial because of the access to innovative treatment and care. These findings reveal that irrespective of health care contexts, what participants in research require, desire, or care about the most is to receive superior and effective treatment and a cure. Similar to Caldwell’s study in Australia, guardians stated that their child would be better monitored when he/she was in a research study [42]. This is likely more influential on decision-making and consent, especially when the local quality and standard of care is lower than the standard received when participating in research, as in many LMIC settings. It is not surprising in a setting like Malawi that high levels of illiteracy [43] and insufficient awareness of human and medical rights [34] may affect understanding of research concepts by guardians. Other studies in LMICs have also reported that the high societal status of physicians prevents patients from questioning their doctors and that poor access to medical services [26] increases the likelihood of being enrolled in a study.

Medical benefits are difficult to define as there are no guidelines on how they should be described in consent forms or during the consent process. The participants may face frustrations if these anticipated benefits are not met during the study [44]. Studies are needed to define the level of ancillary care in this setting and how it should be incorporated in the consent process.

The danger of undue inducement also needs to be considered. On a similar note, we recommend that research staff should ensure that privacy is maintained during individual consent rather than taking place in front of others, as reported in this study, to prevent peer influence which could also lead to undue inducement. However, it is important for staff obtaining informed consent to clearly state that refusal to participate will not jeopardise access to standard of care. Although this is unlikely in acute settings where the child is severely ill, the desire for enhanced care is likely to always dominate decisions on consent and encourage parents to pay less attention to risks; especially when the standard of care is perceived as suboptimal, as is often the case in LMIC settings [17, 24, 45].

Furthermore, poor and inaccurate recall of medical information provided is often related to the patient’s age and levels of anxiety [46]. Patients tend to focus on diagnosis-related information and fail to register instructions on treatment [46]. Our participants were given information at a time when their children were sick, and it is highly likely that their anxiety might have affected retention and recall of information. A study in Malawi which explored the effectiveness of rectal suppositories in children with moderately severe malaria, reported that parents with severely ill children struggled to comprehend large amounts of information due to the stress they felt about their child’s condition [31]. Participants need adequate information about the study to give informed consent. Omitting some of the information might compromise the credibility of the study and the ability of participants to make an informed decision, but there may be ways of focusing the key information so as not to overwhelm guardians at a stressful period. Re-consenting at a later period may also help with this.

### Culture

Despite differences in household hierarchies between ethnic groups in Malawi, either patrilineal (wife lives in husbands home) or matrilineal (husband lives in wife’s home), men are generally still the main decision-makers in Malawi regarding the health care of the family and household decisions in general [47]. In patrilineal societies, the husband and his relatives are the main decision makers while in matrilineal societies, the wife’s brothers are the main decision makers. In contrast to the underlying cultural gender norms, this study identified that mothers or female guardians who had accompanied the child to the hospital generally made the decisions in acute and emergency settings. As a result, this decision-making practice bears the risk of causing conflict in families. However, this was different from non-acute settings where female guardians had a chance to consult their families or spouses before making decisions. Similarly, research staff from acute settings from both clinical trials and observational studies faced some challenges in consenting mothers in the absence of their partners. Respecting the underlying gendered decision-making process often resulted in low recruitment numbers of participants.

The study findings confirm the important role that cultural background plays in health care research. In many African contexts, individuals decide important issues in consultation with family, friends, and community members. This approach is related to an Ubuntu perspective [48], where decision-making with regard to medical interventions is often based on collective rather than individual processes [48]. In contrast, an individualistic approach to decision-making in Western culture is based on the concept of individual autonomy, where choices and actions are based on personal beliefs and values. [49]. This approach clashed with some guardians as they felt uncomfortable that their cultural perspective was not respected. Balancing the scale between individual consent and cultural dynamics in order to improve ethical conduct in research requires careful consideration. In some communitarian cultures, many do not necessarily prioritise the principle of individual autonomy because the emphasis is on communal decision-making, which is perceived as in the best interest of the group, family or society. Studies that have taken this approach into account in the Malawian setting and have pursued a collective consent approach have been successful [50].

Guardians in this study also suggested community sensitisation as a key activity that each study must consider before participant recruitment in this setting. These findings are manifestations of how culture heavily influences the informed consent and decision-making process and differences between African and western cultures need to be understood by researchers and ethical authorities. Researchers in this context must have skills in understanding, appreciating cultural differences and similarities and working with and respecting such diversity during the consent process [51]. More studies are needed to understand how best these two perspectives could be incorporated into the informed consent process without compromising the human rights of individuals and vulnerable groups, especially within the context of acute settings where timely decisions must be made.

### Research process

Adequate information and effective communication from research staff are key in the consent process. The findings in this study have shown that it is not always the case that the same sets of principles and procedures on informed consent are equally applicable to research among different groups. With any research, it is necessary for research staff to consider how much information to give during consent, how to give such information, when and how often to give it. When gaining an informed consent, consider vulnerable groups that might be affected with the way informed consent is obtained and consider the types of research that might need different approaches [52].

Consent is considered voluntary if it is given without any internal or external influence [21]. In this study the voluntary nature of participation was not well narrated to participants. While research staff recognised their obligation not to force the participants, they acknowledged limits to their explanations due to the pressure to meet study targets. Similarly, a study on assessing quality of informed consent in Uganda found that a third of participants were not aware that they could withdraw their participation at any time even though all the studies selected had incorporated this section in their consent forms [16].

In this study, most participants from the clinical trial, acute setting, and observational studies from both acute and non-acute settings, were unable to recall specific information that had been communicated to them, particularly that relating to risks and randomisation. This is not surprising, as other studies [9, 32] have reported that participants’ understanding depends on health literacy as well as on the duration of the informed consent process and the skills of the researchers seeking consent. Participants who failed to narrate specific information tended to report that they were not given information leaflets (or had not read them if given), had forgotten the information provided, had received inadequate time and explanations, or cited inappropriate timing of consent and the urgency of their child’s condition as contributing to their inability to recall information. This is in line with other studies indicating that patients’ lack of comprehension of presented information about the research is an obstacle to informed consent, which may be due to unfamiliar terminology, the hospital environment, cultural and language barriers, and low literacy levels [2, 8–10]. Participants who have given their consent often do not fully understand their rights as participants and a significant proportion of them do not remember consenting to participation on their own or on behalf of their child [11].

It is well known that participants from LMICs and those with low levels of literacy are less likely to understand some aspects of study information during consenting like randomisation, voluntariness, risks, and the right to withdraw [12, 13]. Other studies have found that not only parents misunderstood these components but also adult patients, adult physicians, and paediatricians [53, 54]. Equally, other studies have reported research staff emphasising issues that they anticipated a participant to understand quickly and downplaying issues that might be difficult for a participant to understand such as study design, randomisation and the selection procedure [29]. The most appropriate time to consent children has been stated as when the child is stable, both in this study, and another conducted in Malawi [17]. The study designs should include adequate time for consent that would prevent research staff rushing through the process. Studies are needed to evaluate innovative ways of obtaining consent in acute settings without compromising study protocols as well as ethics guidelines on the practice of delayed consent process.

Despite efforts to communicate clearly to prospective study participants, it remains a challenge in Sub-Saharan Africa [9, 55]. Recommendations have been made for improving understanding of consent information among participants in resource-limited countries. Corneli et al. [27] demonstrated how formative data were used to develop culturally appropriate counselling cards specifically for trials in Malawi to improve understanding of study information [27]. Other researchers have recommended participants’ information being translated into the local language to enable comprehension of the research and health terms used in research [56]. This was done in the research projects investigated in our study. However, our study has shown that there are still challenges in understanding study information across all study settings and types.

The use of study information leaflets during consenting is universally recognised and is essential to ensure that research participants understand the aims and risks of the study and can voluntarily consent to participation. However, studies have reported that research participants often do not understand the content of the information sheet or the consent form for the study, especially if the consent form is lengthy and includes unfamiliar wording [57, 58]. Some participants may understand information at first contact while others may need to go through the information many times, although some female guardians did not show interest in taking information sheets. However, in the Malawian setting it is mostly women who nurse their sick children. Therefore, there is also a need to design information sheets appropriate for the study setting and study population to facilitate reading, comprehension, and retention.

Our sample contained participants across different study settings and types. We chose to include a wide range of studies to do a comparative analysis of the consenting process. However, we were limited due to the number of studies that met our inclusion criteria.

## Conclusion

Our study describes the views of guardians of children and research staff involved in paediatric research about the process of consent in different study designs and settings in Malawi. Despite academic consensus about the importance of informed consent, we noted variations in the understanding of study information by guardians.

We found that the motivation to participate in the study, the decision-making process and the delivery of study information varied across study designs and settings. The causes of variations included inequalities in healthcare quality between research ward and government hospitals and cultural/gender roles in making decisions about children. We also noted that research staff might feel pressure to recruit participants and tailor the consenting process to achieve this. More guidance is needed on how these factors can be addressed during the consenting process, so that consent for future studies is more fully informed. Furthermore, the informed consent process should have a degree of flexibility depending on the study design and setting for participants to understand the study information better.

We recommend training for research staff involved in the consenting process. In response to the study findings, MLW, through its Clinical Research Support Unit (CRSU), implemented Informed Consent training in 2018, and a study could now evaluate if the informed consent has improved. The use of one size fits all consent process may not be ideal. The consent process should be informed by the study design and setting. In addition, we recommend a two-step consent approach for acute settings which will include (1) initial consent to provide standard care (2) once a person is stable, full consent for the study to be done. This process will promote understanding as guardians are more likely to concentrate on study information when their child is in stable condition. Future research is recommended to test innovative and interactive informed consent material to promote understanding of informed consent. Furthermore, research on detailed suggestions on how husbands could get involved in the consenting process, especially for acute settings, is essential. Further research to identify ways on how to improve the consenting process, comprehension of research information by guardians and information giving by research staff in acute settings, especially in low-income countries is essential.

## Supplementary Information

**Additional file 1.** Interview guide for guardians and research staff.

## Data Availability

The datasets used and/or analysed during the current study are de-identified and available from the corresponding author on reasonable request.

## Abbreviations

CIOMS: Council for information organisation of medical sciences
HDU: High dependency unit
OPD: Outpatient department
MLW: Malawi-Liverpool-Wellcome Trust, Clinical Research Programme
QECH: Queen Elizabeth Central Hospital
COMREC: College of Medicine Research Ethics Committee
SIOP: International Society of Paediatric Oncology
CRSU: Clinical Research Support Unit
