# Supplementary material for: Guardians and research staff experiences and views about the consent process in hospital-based paediatric research studies in urban Malawi: A qualitative study
Source: BMC Med Ethics. 2022 Dec 5;23:125. doi: 10.1186/s12910-022-00865-x (PMC9720930; doi:10.1186/s12910-022-00865-x)
Supplement: Supplementary file 1 [file 12910_2022_865_MOESM1_ESM.odt]

ADDITIONAL FILE 1: INTERVIEW GUIDE FOR GUARDIANS AND RESEARCH STAFF 

Appendix 1:  In-Depth Interview Guide for Guardians

Section 1: the following questions will answer objective 1 of the study, which is to examine participant understanding of study information at enrolment and one month post enrolment 

- How did you get to know about the study that your child is enrolled in? (Probe on the following: how the study team interacted with them, who contacted them, when, where?)

- What is the study that your child is in, what does the study involve and their role? (Probe on the following: understanding of study, why their child was chosen,  risks, benefits, voluntariness, right to withdrawal anytime, expectations/compliance with study procedures, whether a copy of patient information leaflet was given to them, free to ask questions and who to contact for more information?)

- For clinical trials only: 

What are the available treatment allocations and to which arm was the child randomised? (Probe on the following: understanding on available treatment arms, the arm child is in, how the concept of randomisation works in the study?) 

  

Section 2: the following question will answer objective 2 of the study, which is

to explore broader participant factors that may influence understanding of

informed consent.

- What factors influenced your understanding of informed consent for the current study that your child is in? (Probe on education background, language, role in community, support, decision making, age, relationship with child, sickness of child, timing of consenting, experience with research, presentation and content of patient information leaflet)

Section 3: the following question will answer objective 4 of the study which is to assess perceptions of participants about mode of administration and timing of informed consent in acute/emergency and in general care for clinical trials in opposition to observational studies. 

- How did you see about the mode of administration of participant information to you to make an informed decision to enroll child in the study? (Probe on the means, and timing of consenting) 

Section 4: the following questions will answer objective 5 of the study which is to explore what participants perceive as good research practices around informed consent

6.       How would you describe good research practices around informed consent? (Probe on satisfaction, approach?)

  Appendix 2: Interview Guide for Research Staff Participants

Section 1: the following questions will answer objective 6 of the study, which is to assess the challenges around informed consent processes faced by research staff involved in obtaining informed consent from potential participants in paediatric research. 

- Describe your experience with obtaining informed consent in your study? (Probe on the following: challenges faced, time, privacy, content, presentation, language, experience, relationship, training, age, gender)
